# Intergenic regions of *Borrelia *plasmids contain phylogenetically conserved RNA secondary structure motifs

**DOI:** 10.1186/1471-2164-10-101

**Published:** 2009-03-06

**Authors:** Nicholas Delihas

**Affiliations:** 1 Department of Molecular Genetics and Microbiology, School of Medicine, Suny, Stony Brook, NY 11794-5222, USA

## Abstract

**Background:**

*Borrelia *species are unusual in that they contain a large number of linear and circular plasmids. Many of these plasmids have long intergenic regions. These regions have many fragmented genes, repeated sequences and appear to be in a state of flux, but they may serve as reservoirs for evolutionary change and/or maintain stable motifs such as small RNA genes.

**Results:**

In an in silico study, intergenic regions of *Borrelia *plasmids were scanned for phylogenetically conserved stem loop structures that may represent functional units at the RNA level. Five repeat sequences were found that could fold into stable RNA-type stem loop structures, three of which are closely linked to protein genes, one of which is a member of the *Borrelia *lipoprotein_1 super family genes and another is the complement regulator-acquiring surface protein_1 (CRASP-1) family. Modeled secondary structures of repeat sequences display numerous base-pair compensatory changes in stem regions, including C-G→A-U transversions when orthologous sequences are compared. Base-pair compensatory changes constitute strong evidence for phylogenetic conservation of secondary structure.

**Conclusion:**

Intergenic regions of *Borrelia *species carry evolutionarily stable RNA secondary structure motifs. Of major interest is that some motifs are associated with protein genes that show large sequence variability. The cell may conserve these RNA motifs whereas allow a large flux in amino acid sequence, possibly to create new virulence factors but with associated RNA motifs intact.

## Background

Intergenic regions of bacterial chromosomes carry important functional units such as transposable elements [1]. Small regulatory RNA genes are also abundantly found in regions between protein coding genes [2-7]. In *E. coli*, many intergenic regions and non-coding strands of known genes are transcribed, resulting in a heterogeneous collection of RNA transcripts, many of which are <65 nt [8]. Bacterial intergenic chromosomal regions also carry numerous small repeat sequences that can fold into RNA-type secondary structures [9-12]. Some represent non-autonomous miniature inverted repeat transposable elements (MITEs) [13,1]. Many are found immediately downstream of, or overlapping terminal codons [14-16] and may be regulatory units [14,15,17]. Small repeat elements carry a variety of motifs at either the DNA, transcribed RNA or translated protein levels and they may be engines for evolutionary change [16,17].

*Borrelia burgdorferi *was first isolated and shown to be the etiologic agent of Lyme Disease in the early 1980s [18,19]. The chromosomes of *Borrelia burgdorferi str. B31 *and its related species, *B. afzelii PKo *and *Borrelia garinii PB*, have been sequenced, as well as many of the associated plasmids [20-23]. These organisms possess multiple plasmids. For example, *B*.*bugrdorferi *strain B31 has 12 linear plasmids and 9 circular plasmids [20,21]. *Borrelia *chromosomes are small relative to many bacterial genomes, e.g., the genome of *Borrelia burgdorferi str. B31 *is ~0.9 Mb and *Yersinia pestis str. Co92 *genome is 4.6 Mb. *Borrelia *chromosomes represent a tight packing of protein genes where there is little intergenic space. On the other hand, plasmids contain a much larger amount of intergenic space. These regions are known to have sequences that translate to repeat units of small peptides. In addition, they contain a high percentage of fragmented genes, including those from transposase genes, and interesting fusions of protein motifs as well as [21]. This shows a rapid evolutionary trend in these regions and perhaps plasmid intergenic regions are where new protein and RNA genes and other functional units may evolve.

A small number of *Borrelia *non-coding RNA genes have been detected [24,25]. It has been assumed that *Borrelia *has few small RNA genes, based on comparative genomic searches for similarities to known bacterial small RNA sequences [24]. However many regulatory RNA gene sequences diverge between species, e.g., *micC, micF *and *ryhB *(see Rfam website [26,27]), and analogous genes in other species can be missed, especially between distantly related species. In some cases, such as the regulatory RNA DsrA, nucleotide sequences from different species show few similarities [25]. Intergenic regions have not been further analyzed for evolutionarily conserved RNA secondary structure motifs. These motifs can signal the presence of functional units.

In a bioinformatics study, we show that several repeat sequences in plasmid intergenic spaces and/or sequences immediately downstream of coding regions sustained multiple mutations, yet these sequences fold into highly conserved RNA-type stem loop structures. Evolutionary conservation indicates an essential role for these structures in the cell. In contrast, super family protein genes associated with some conserved RNA-type structures display marked amino acid and peptide chain length differences and appear to be in a process of change and/or decay. This raises interesting questions concerning how these peptide-RNA linked elements will evolve with time.

## Results

Repeat sequences of intergenic nucleotide sequences of *Borrelia *plasmids were analyzed for secondary structure motifs using the Zuker m-fold program [28,29]. In addition, the RNAz program was used to confirm thermodynamically stable and evolutionarily conserved RNA secondary structures [30]. Intergenic sequences from plasmids lp60 and lp28 of *B. afzelii Pko *were completely scanned manually for repeat sequences and RNA motifs. In addition, selected regions that contain relatively large intergenic regions from *B. burgdorferi B31 *and *Borrelia garinii PB *plasmids were also scanned. Most regions did not yield conserved stem loop structures, however five intergenic nucleotide sequences were found to display evolutionary conserved stem loop structures (Table 1).

**Table 1:** Nucleotide sequences that display secondary structure features

Species	Plasmid	Positions	Sequence
SEQUENCE #1			
*B. afzelii PKo*	lp25	573–632	ATAACAAAGAATTCTCCACC
			TATAATTTCTATGAAATTTAG
			GTGGAGATGAATTTGTTAA
			
SEQUENCE #2			
*B. afzelii PKo*	lp34	1711–1804	TAAAAGCATATCTTTTA
			TTAAAGATATGCTTAAT
SEQUENCE #3			
*B. afzelii PKo*	lp60	50661–50585	ATACTAAATAAACAAAAAATT
			AATACGTTGCACTTTATATTT
			TTTAAAAAAGAGAAGTTAATT
			CTTCTCTTTTTTTT
SEQUENCE #4			
*B. afzelii PKo*	lp60	26239–26360	ATTGGGTTTAAAACTACA
			AATAGGGCCTTAAGGCC
			CTATTTGTAGTTTTAAAGA
			AGTTTTCAATGAATTGTTA
			ATTTATAACAATAAACAAGT
			ATATATCTCACTATAGTTT
			TTTTCAAATA
SEQUENCE #5			
*B. burgdorferi B31*	*lp54*	14820–14969	AATATTTATTTGCAAAACTT
			GAAAAGTTAGTGTATACTTT
			ATAGGTACAGACTGACACGC
			AATGTGTCGCTCTTAATATAA
			GGACCTGTTACCTTAAAGGGT
			TTATTGGGGATTCTTTTAAAA
			GAATCCCCAATAAACCCTTTA
			ACTTTT

### Sequence #1

**Figure 1:**
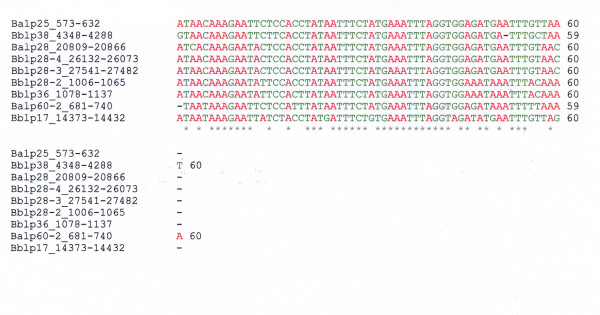
**Alignment of plasmid nucleotide sequences related to *B. afzelii PKo *lp25 Sequence #1**. Adenosine residues are colored red, all other residues are green. Color scheme is for ease of viewing only. The EMBL-EBI CLUSTALW 2.0.8 multiple sequence alignment program [31][32] was used for alignment. Numbers adjacent to plasmid names refer to nt positions in plasmid sequences. A star (*) denoted invariant positions.

A 60 nt intergenic sequence (Sequence #1, Table 1) was found in nine plasmids from *B. afzelii Pko *and *B. burgdorferi B31*. Alignment of these sequences reveals a major conserved region that is approximately at the center of the polynucleotide nucleotide chain (Figure 1). The EMBL-EBI CLUSTALW 2.0.8 multiple sequence alignment program [31,32] was used for alignment. Twenty out of 60 nucleotide positions show base substitutions. A comparison of sequences shows a 77–100% sequence identity between the nine plasmid sequences. Sequences homologous to Sequence #1 have not been detected in *B. garinii PBi *plasmids or *Borrelia *chromosomal sequences.

**Figure 2:**
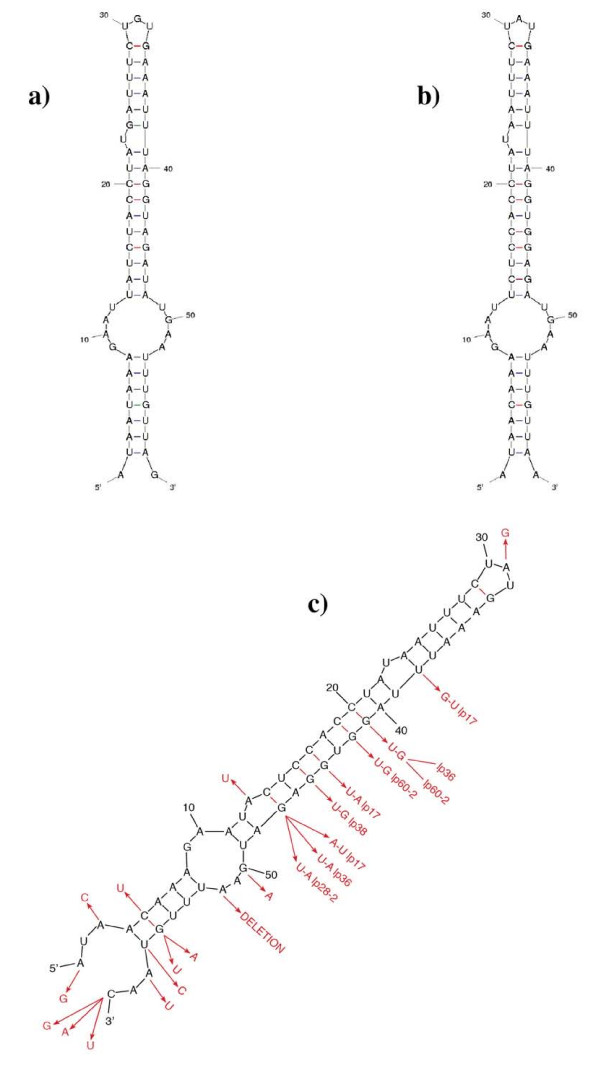
**RNA secondary structure models of Sequence #1 nt sequences from a) Bb lp17 b) Ba lp25 and c) Bb lp28-4**. The mfold (version 3.2) program by Zuker and Turner [28,29] was used for secondary structure modeling. Figure 2c shows base substitutions (marked with red arrows) that are found in all positions in the nine plasmid sequences.

RNA secondary structure modeling of the nine sequences shows a high conservation of secondary structure with multiple base substitutions that maintain base pairing. In addition, a bulged U at position 23 is found invariant in all nine sequences. Figure 2a–c depicts RNA secondary structure models from three of the nine plasmid sequences. Base substitutions at individual positions are depicted in Figure 2c. Mutations at six base-paired positions in the upper portion of the stem loop show compensatory changes that conserve the stem structure (Figure 2c). Prominent are the base pair changes at positions C_14_-G_47 _that result in A_14_-U_47 _pairing in the sequence of plasmid Bb pl17 (Fig. 2a) and U_14_-A_47 _pairing in two other plasmid sequences (Figure 2c). Base pair positions 14 and 47 appear to be "hot spots" for mutations, but nevertheless, Watson-Crick base pairing is maintained. The C_14_-G_47_→A_14_-U_47 _substitution is highly significant in that it shows the double mutation, pyrimidine→purine, purine→pyrimidine. This is a transversion and has a lower probability of occurring than purine→purine and pyrimidine→pyrimidine transitions. The C-G→U-G transition at positions 19,20 and 41,42 (Figure 2c) are between orthologous genes in Bb lp28-4 and Ba lp60-2. The base pairing at the terminal end of the stem differs between several plasmid structures (e.g., compare Figure 2a and 2b with 2c). Although the three A-U base pairs at the base of the stem (positions A_6–8 _and U_53–55_) are conserved in all plasmid sequences, in plasmids Bb lp28-2 and Bb lp36, a G_56_→A_56 _substitution appears to partially destabilize the base stem structure (data not shown). The cut off at the 60 nt length for Sequence #1 was made because sequences extended from the 5' and 3' ends do not yield additional conserved secondary structure motifs. This however does not preclude that the 60 nt stem loop is part of a larger functional unit that may not show prominent conserved secondary structure motifs.

Sequence #1 was also analyzed by the RNAz bioinformatics program [30], which predicts RNA structures that may be evolutionarily conserved. The results as depicted on the RNAz website are shown in Additional file 1. The secondary structure displayed at the bottom of Additional file 1 is identical to that depicted in Figure 2c. The descriptive section at the top of the figure reveals a mean z-score of -6.43 (a score less than 0.0 indicates that a structure is more stable than one expected by chance). The prediction is that Sequences #1 represents evolutionarily conserved RNA structures. Base pairing for five individual sequences is shown in the middle section of Additional file 1. Thus the RNAz analysis confirms the predicted evolutionary conservation of Sequence #1 secondary structures derived manually.

In terms of nearest neighbor genes, the 60 nt repeat element is not located upstream or downstream of plasmid annotated genes in a consistent manner, both in terms of spacing and type of gene, although it is close to two putative transposase genes. For example, the repeat element is found 39 bp upstream of locus BAPKO_4522 in Ba lp28. This locus encodes a putative 378 aa transposase. In Bb lp28-4, it is situated 37 base pairs downstream of locus BB_I41, which encodes a putative 80 aa protein. BB_I41 is a fragmented gene and shares the 5' end of a 155 aa transposase encoded by BB_H40 in Bb lp28-3. In Bb lp28-2, the 60 base pair repeat sequence overlaps the 3' end of BB_G01 by 3 bp. Locus BB_G01 encodes a 297 aa hypothetical protein.

### Sequence #2

A second set of repeat sequences displays inverted repeats and these are found in eleven loci in ten plasmids from the three *Borrelia *species, *B. burgdorferi*, *B. afzelii *and *B. garinii*. Alignment of nt sequences reveals there are a significant number of base substitutions as well as insertions/and or deletions [see Additional file 2]. Nucleotide positions 4–31 (sequence numbering positions from Ba lp34) comprise inverted repeats.

RNA secondary structure modeling of putative RNA transcripts shows that all eleven sequences display stem loop structures which contain 11–13 base pairs. Additional file 3 shows representative secondary structures and depicts several base-pair compensatory changes in the stem, e.g., A_8_-U_27 _pairing in *B. afzelii *lp34 changes to C_8_-G_29 _in *B. burgdorferi *lp25 and A_5_-U_30_→U_5_-A_29 _in *B. burgdorferi *lp28-3. Again, these are examples of pyrimidine→purine and purine→pyrimidine mutational transversions, and these are found between orthologs. Additional base substitutions in other plasmid sequences result in G-U non-canonical pairing (data not shown). The presence of non-canonical pairs implies that the conserved structure may function at the RNA level as opposed to the DNA level. The loop structure sustained base substitutions and insertions/deletions, which resulted in major differences in loop sequences [see Additional file 3]. The stem length varies, but the invariant A_4_-U_31 _pair is always at the terminal end of the stem and is straddled by invariant A_3 _and A_32 _(numbering position relative to the Ba lp34 sequence [see Additional file 3]. This arrangement is found in all eleven of the stem loop structures (data not shown).

By bioinformatics methods, random mutations were introduced in Sequence #2 to ascertain the probability of compensatory base pair changes arising by random base changes. For example, after adding 3 mutations to the 34 nt Sequence #2 and initiating 30 trials of random mutagenesis, the stem was found disrupted (with mispairs) in >90% of trials, and all 30 trials showed a resultant decrease (towards [+] side) in delta G, and in some cases there was a decrease by a factor of 10 in the delta G value. The configuration of the stem was drastically altered in 10 of the 30 trials (data not shown). Single base compensatory changes in the stem occurred in about 10% of trials, but at the same time the accompanying mutations (again, 3 mutations/34 nt were induced) caused a partial disruption of the stem. Double compensatory mutations, such as U-A → C-G and the less probable transversion, U-A → G-C, did not appear. These trials show a trend towards disruption of an ordered structure by addition of random mutations. In sharp contrast, biological mutations within 11 homologous sequences (23 positions showing mutations out of ~34 nt of Sequence #2) display numerous base-pair compensatory changes, including transversions, show no mispairing, no stem alterations (such as formation of a bulged or looped positions), and several insertions/deletions that were closely confined to the unpaired looped region where they do not induce changes in the stem loop configuration. Strong evolutionary pressures appear to maintain the secondary structure motif of Sequence #2.

**Figure 3:**
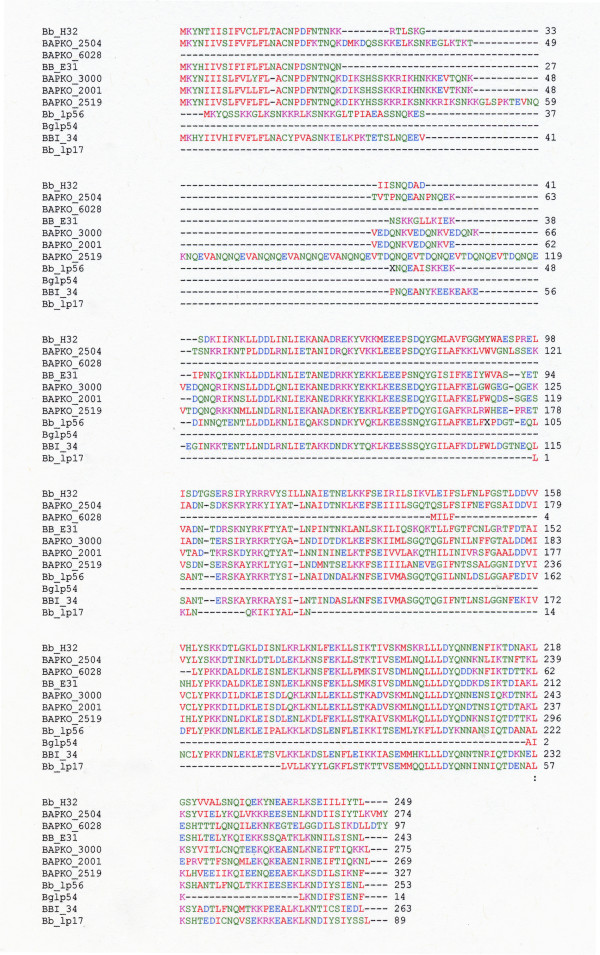
**Alignment of amino acid sequences that have similarities to lipoprotein_1 family proteins, which are linked to Sequence #2**. Peptide sequences are from eleven loci in *Borrelia *plasmids. Sequences identified by plasmid names are those that have not been anotated. X denotes a stop codon found at positions 38 and 98 of Bb lp56. Amino acid color code: red, hydrophobic and aromatic amino acids, blue, acidic, magenta, basic, green, hydroxyl and amine containing as specified by the EMBL-EBI CLUSTALW 2.0.8 multiple sequence alignment program [31,32].

*Borrelia *plasmids contain the superfamily of protein genes that encode *Borrelia*_lipoprotein_1 [20,33,23]. Significantly, the eleven stem loop sequences are found primarily between 14 and 33 bp downstream of a family of lipoprotein_1 genes, as well sequences that encode fragments of lipoprotein_1. The stem loop-associated lipoprotein_1/lipoprotein_1 fragment amino acid sequences are shown in Figure 3. The stem loop sequence in Ba lp60 is 24 bp downstream of locus BAPKO_2001, a putative lipoprotein_1 gene encoding a 237 aa protein. On the other hand, there is no lipoprotein_1 gene annotated upstream of the stem loop repeat element in Bb lp56. This upstream region has high nucleotide and amino acid sequence identities to lipoprotein_1, but the translated lipoprotein_1 amino acid sequence contains several stop codons (Figure 3). With plasmid Bg lp54, the stem loop repeat element is located 93 bp from the left end (5' end) of the plasmid. Thus, most of the upstream sequence of the lipoprotein_1 gene would have been lost upon a putative translocation of lipoprotein_1. Nevertheless, a C-terminal 14 aa fragment is found that is highly similar to lipoprotein_1 C-terminal sequences (Figure 3). The Bg lp54 34 nt stem loop is 26 bp downstream of the 3' end of the sequence encoding the 14 aa C-terminal fragment. On the other hand, there is an 18 bp overlap of the stem loop sequence with the 3' end sequence of locus BB_H32 encoding a lipoprotein_1 gene in Bb lp28-3, yet the stem loop structure is conserved with multiple base-pair compensatory changes (e.g., compare figures a and d, Additional file 3). Thus in cases where the stem loop has been detected, there is phylogenetic conservation of secondary structure and conservation of its location downstream of or partially overlapping lipoprotein_1 genes/lipoprotein_1 gene fragments. The high conservation of repeat element secondary structure suggests there is evolutionary pressure to maintain a subset of lipoprotein_1 genes/gene fragments with downstream sequences that can fold into a stem loop structure. However, not all lipoprotein_1 genes have the conserved hairpin and the stem loop may be specific to the subset of lipoprotein-1 genes outlined above. For example, locus BAPKO_4514 in Ba lp28 encodes a putative 261aa lipoprotein_1, which does not have the characteristic stem loop.

### Sequence #3

*Borrelia *sp. encode the virulence factor termed complement regulator-acquiring surface protein 1 (CRASP-1) [34-36]. This protein binds factor H, resulting in inhibition of complement activation in mammals. CRASP-1 proteins from *B. afzelii *lp54 and other *Borrelia *species plasmids have been isolated and their properties characterized [36].

Multiple copies of sequences analogous to CRASP-1 genes have been detected in *Borrelia *plasmids. These include sequences in loci BAPKO_2065- BAPKO_2070 from *B. afzelii *lp60 [see Additional file 4]. A comparison shows that the translated aa sequence from Ba lp60 locus BAPKO_2068 and the aa sequence derived experimentally from the Ba lp54 CRASP-1 protein (whose gene locus is termed *Ba_lp54 mmsa71*) are almost identical and show only 5 aa changes out of 241 aa. In addition, the factor H binding motif, _232_KDLDSFNP_239 _is present in locus BAPKO_2068 and the Ba lp54 CRASP-1 *mmsa71 *gene [see Additional file 4]. BAPKO_2068 and *Ba lp54 mmsa71 *(CRASP-1) probably are paralogous genes and the BAPKO_2065-2070 superfamily are also paralogs resulting from gene duplication. However an amino acid sequence alignment of this family of loci shows several major insertions/deletions and amino acid substitutions [see Additional file 4]. The bottom figure in Additional file 4 shows a phylogram of this gene family.

**Figure 4:**
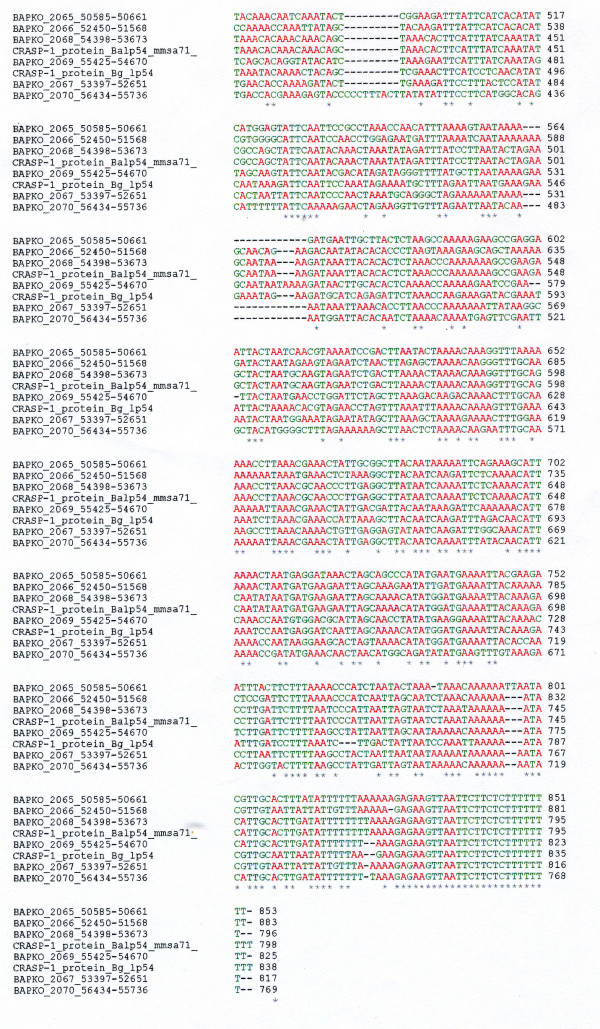
**Alignment of 3' segment of nt sequences from CRASP-1 in Bg lp54, CRASP-1 in Ba lp54 and CRASP-1-related loci BAPKO_2065-2070**. Adenosine residues are colored red, all other residues are green. Colors are for ease of viewing. The EMBL-EBI CLUSTALW 2.0.8 multiple sequence alignment program was used. A star (*) denoted invariant positions.

Alignment of nucleotide sequences immediately downstream of open reading frame stop codons from BAPKO_2065 to BAPKO_2070 and CRASP-1 genes from Bg lp54 *zqa68 *and Ba lp54 *mmsa71 *(reference position, TAG_726 _BAPKO_2068) shows that these sequences are highly conserved (Figure 4). Secondary structure modeling of putative transcripts of downstream sequences show two stem loop structures [see Additional file 5]. Stem loop #2 is highly conserved with numerous base pair compensatory changes, but it also has a 3' terminal oligouridine. It probably represents a Rho-independent transcription termination site for CRASP-1 and related putative gene transcripts. Stem loop 1 is present in all repeat sequences but shows variations in secondary structure (e.g., compare figures a and b in Additional file 5). The significance of this stem loop is unknown, but it may reside within a putative 3'UTR region. The high conservation of the stem loop 2 secondary structure contrasts with the variability in overall amino acid sequence, differences in factor H binding site sequence (_232_KDLDSFNP_239_) and peptide chain length of associated protein genes [see Additional file 4].

**Figure 5:**
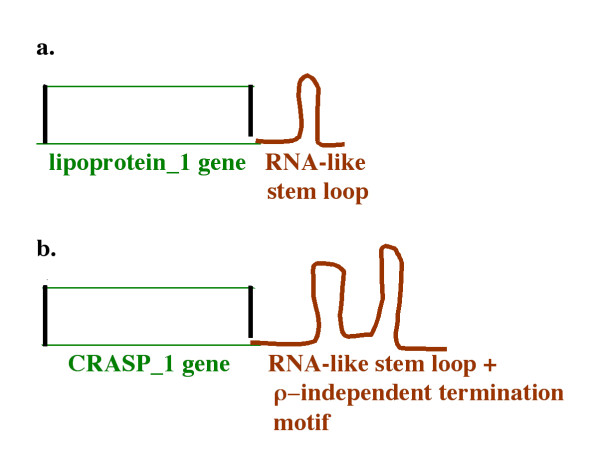
**Diagrammatic representation of lipoprotein_1 and CRASP-1 genes with conserved RNA structures linked to the 3' ends of the genes**.

Figure 5 shows a diagrammatic representation of virulence protein genes lipoprotein_1 and CRASP-1 with their associated RNA motifs at the 3' ends. The amino acids sequences of both genes vary between homologous sequences and some gene copies are degenerate. However, the associated RNA secondary structures are evolutionarily highly conserved.

### Sequence #4

Sequence #4 (Table 1) is 122 nt and has an inverted repeat segment (positions 26245–26290 in Ba lp60). This sequence is not located near any protein genes. It is 233 bp downstream of locus BAPKO_2033, which encodes an oligopeptide ABC transporter, and 171 bp upstream of BAPKO_2034, a putative lipoprotein gene. Sequence #4 from Ba lp60 is highly conserved in Bg lp54, but only a part of the sequence is conserved in Bb lp54 [see Additional file 6]. Comparison of positions 52–122 show less than 40% similarity between sequences of Bb lp54 and Ba lp60. A comparison and analysis of Sequence #4 and flanking regions in plasmid Bb lp54 shows that the 122 bp sequence has been recombined in the opposite orientation (data not shown). This resulted in a major change in sequence between Bb p54 and Ba lp60 and Bg lp54 downstream of position 53 [see Additional file 6]. The approximate 5' half of the sequences are very similar due to the inverted repeat, which provides a similar sequence in the opposite orientation.

Secondary structure models of the 122 nt sequence reveal that a structural motif is conserved between the three sequences [see Additional file 7]. The three models display two stem loops and one small stem (stem 3). Stem loop 1 consists of the inverted repeat and has 21 contiguous Watson-Crick base pairs (positions 7–52 in Ba lp60). The sequence in Bb 54 is not a perfect inverted repeat but there are base compensatory changes that maintain the 21 Watson-Crick base pairs, i.e., there are two G-C pairs in Bb lp54 in place of two non canonical G-U pairs in Ba lp 60 and Bg lp54. The presence of stem loop 2 is of major significance in that it is in a region with very low nt sequence identity, yet a similar stem loop is maintained but with extensive differences in base pairing (compare stem loop 2, figures 1a and 1b, see Additional file 7). This type of phylogenetically conserved motif is characteristic of secondary structural properties of some small non-coding RNAs, where secondary structure and not necessarily sequence is conserved, e.g., see [37]. This conservation implies a functional importance for this 122 nt sequence, which may represent a non-coding RNA.

### Sequences #5

Sequence #5 is 150 nt in length and is found in plasmids of three *Borrelia *species, Ba lp60 from *B. afzelii PKo*, Bb lp54 from *B. burgdorferi str. B31 *and Bg lp54 in *B. garinii PB*. This sequence is highly conserved with nt sequence identities > 95% between the three sequences. The sequence at positions 14913–14964 from *B. burgdorferi B31 *plasmid lp54 represent a perfect inverted repeat. Secondary structure modeling shows the presence of three stem loops in all three plasmid sequences. The structure from Bb lp54 is shown in Additional file 8. It is highly stable thermodynamically with a delta G of -53.3 kcal/mol. A comparison of different plasmid structures shows three base substitutions in stem 3 that maintain the base-pairing, but two other substitutions produce A-A and A-G non-canonical pairs (data not shown). Stem 3 is not destabilized, but there are small decreases in the delta G value to -43.3 kcal/mol (Bg lp54) and -43.6 kcal/mol (Ba lp60). In the context of surrounding base pairs, non-canonical pairs such as A-A and A-G are present in RNA double helices [38] and have been found to contribute to RNA stem double helical conformations [39,40].

An analysis of Sequence #5 by the RNAz bioinformatics program shows a predicted evolutionarily conserved RNA structure with a mean z-value of -6.04 [see Additional file 9].

Sequence #5 is immediately downstream of loci BAPKO_2021, BGA_19 and BB_A21. These loci represent highly conserved proteins that have been annotated as hypothetical proteins. However they have amino acid sequence and putative protein domain similarities to a plasmid partition protein (PF-49 encoded on plasmid cp32-11 in *B. burgdorferi*) when analyzed by Pfam [41-44]. The aa sequence identity to PF-49 is 55% as determined by the ExPASy Proteomics Server [45,46]. Although the Sequence #5 stem loops reveal an interesting highly stable structure that is linked to a conserved protein, additional sequences homologous to Sequence #5 would be needed to further support a proposed phylogenetic conservation of secondary structure.

## Discussion

In genomes of many bacterial species, intergenic regions are found to be rich in repeat elements such as MITEs [9,12,13,1,17], other small nucleotide sequence repeats [11,47] and small non-coding RNA genes [48,3-7]. Here we analyzed intergenic plasmid regions from three species of *Borrelia *and have detected intergenic sequences that can fold into conserved RNA secondary structures. Compelling evidence for evolutionary conservation comes from comparisons of homologous sequences, where numerous base-pair changes are found to maintain stem loop structures. These stem loops are specific to plasmid sequences, and none have been detected in *Borrelia *chromosomes or in sequences from other bacterial species.

Two RNA-motifs associated with super families of protein genes (lipoprotein_1 and CRASP-1) show a high conservation of secondary structure between homologs, yet these gene families show extensive amino acid substitutions and deletions/insertions. Perhaps the cell maintains these RNA motifs as reservoirs and as potential functional units in the formation of new variant proteins. A major focus in future work should be to determine if variant CRASP-1 and lipoprotein-1 loci are translated.

Sequence #2 contains inverted repeats and is located less than 35 bp downstream of putative lipoprotein_1 genes, and in one case overlaps the terminal codon sequences. This is very similar to the location of several miniature inverted repeats, the MITEs that are present in other bacterial species. These inverted repeats are also found downstream of genes, and in some cases are found to overlap C-terminal codons [13,14,16,17,49]. In *Yersinia*, genes situated upstream of MITEs appear to be regulated by these inverted repeat elements, which are transcribed into RNA [50]. Although Sequence #2 differs from bacterial MITEs in not having a large nucleotide segment between inverted repeats, the proximity of this sequence to C-terminal coding ends of genes is similar to that of several MITEs.

*Borrelia *contains transposase genes that are found in other bacterial species [20]. Some plasmids show a high percentage of transposase-specific nucleotide sequences which may not be evident from gene annotations, e.g., the first ~1400 bp of the left side of *B. afzelii PKo *plasmid lp28 starting at nucleotide position 1 consists entirely of transposase-related sequences (unpublished results). There may also be non-autonomous transposable elements present in *Borrelia *that are moved and replicated by transposases. As many other bacteria contain these elements [1], it would not be surprising if *Borrelia *had its own set of non-autonomous small transposable elements, possibly with their own specific signatures. Repeat Sequence #2 described above should be further analyzed for a possible relationship to bacterial MITEs.

Stem loops that are proximal to protein genes have been reported before. Dunn et al [51] described two inverted repeat sequences in tandem with perfect base paired stems in *B. burgdorferi *in circular plasmid cp8.3. The hairpins are adjacent to putative -35 promoter sequences of an open reading frame. Also, an inverted repeat sequence is found in the 5' flanking region of the bba64 (P35) gene in *B. burgdorferi *[52]. However the above sequences, which are upstream of genes in promoter regions, are unrelated to those reported here.

Stem loop 2, from Sequence #3 is downstream of the CRASP-1-related genes and appears to have classic Rho-independent termination signatures in terms of size and oligo U tail. The adjacent stem loop 1 may be part of a putative 3' UTR of CRASP-1 and CRASP-1-related proteins. Functions can not presently be assigned, but it should be noted that some small RNAs in *E. coli *represent 3' UTR transcripts which show different expression levels from associated mRNAs and may have independent functions [8]. Sequences #1, #4, and #5 appear to have typical RNA signatures with long stem loops and bulged/looped positions. Without further characterization, functional roles cannot be assigned. But of particular interest is the conservation of the bulged U at position 23 of the Sequence #1 stem loop. Many RNA secondary structures display conserved bulged positions and these have functional roles in RNA/RNA interactions [53,54]. Sequence #1 does not appear to be linked to any protein genes and is present in nine different plasmids. This poses the question of how it was transferred and why the sequence is duplicated. Interestingly, Sequence #4 is found in three different species, *B. burgdorferi str. B31*, *B. afzelii PKo *and *B. garinii PB *but in only one copy number. Thus this RNA motif may provide an essential function in *Borrelia*, as it is found in all three species. Once complete genome sequences of other *Borrelia *species are determined, it would be of interest to see if Sequence #4 and/or its characteristic secondary structural model is also present in these species.

Only a limited number of plasmids have been analyzed for repeat sequences that fold into RNA motifs, but a more comprehensive search is necessary to assess their abundance. Experimental RNA analyses such as Northern blots needs to be done to determine if these sequences are transcribed, but in view of the strong evidence for evolutionary conservation of secondary structure, they may function at the RNA level. In *E. coli*, many intergenic sequences are transcribed, which results in the presence of a large number of heterogeneous small RNAs [8]. These elements also have not been analyzed for function.

## Conclusion

Small repeat sequences of *Borrelia *sp. linear plasmids show numerous changes in nucleotide sequence, nevertheless, RNA-type motifs generated by these variable sequences are highly conserved evolutionarily. Two of the motifs may be candidates for non-coding RNAs. Two others appear linked to C-terminal ends of super families of protein genes/pseudogenes, but these genes display major changes in amino acid sequence and peptide chain length. Jacob Monod described evolutionary change in terms of "tinkering", a trial and error process in formation of new or modified genes with random mutations and/or random fusion of motifs [55]. Perhaps the variable super family virulence protein gene sequences show elements of "tinkering", however the interesting question is why the RNA motifs, which have also sustained mutations are well conserved when at least some of the associated protein genes are in a process of change or decay. We have mentioned the possibility of these being reservoirs for formation of variant or new proteins.

## Methods

To search for conserved intergenic sequences, NCBI/BLAST BLAST Assembled Genomes [56] and BLAST with microbial genomes [57] were used. Blast with microbial genomes used a value of 10 for expect and the default filter. Nucleotide blast searches were optimized for both highly similar sequences megablast and discontiguous megablast. Default parameters were used. For similar sequence megablast the parameters were: maximum target sequences, 100; automatically adjusted for short sequences; expect, 10; word size, 28. Discontinuous match/mismatch scores, 1,-2; gap costs, linear; filter, low complexity regions. Discontinuous megablast: same parameters as those of similar sequence megablast with the exception word size, 11; match/mismatch scores, 2, -3; gap costs, existence: 5 extension: 2.

The Swiss Institute of Bioinformatics SIB ExPASy Blast server [46] was used to find protein homologies. The blast program and data base used was: blastp – query against the UniProt Knowledgebase (Swiss-Prot + TrEMBL) and default parametes as shown under "Options" were used. The database was the complete database.

Initial searches for repeat sequences and RNA motifs were performed by "walking" intergenic sequences from plasmid lp28 of *B. afzelii Pko*. In addition, several regions that contain relatively large intergenic sequenes from *B. burgdorferi B31 *and *Borrelia garinii PB *plasmids were also scanned.

Intergenic regions were scanned at 200 bp at a time for conserved or partially sequences. These sequences were then modeled for conserved RNA stem loops. Cut offs in regions 5' and 3' of a determined stem loop(s) were made when the additional sequences failed to provide conserved stem-loops. Reverse transcript sequences as well as overlapping sequences at the 200 bp junctions were also structure modeled. Repeat sequences were found that displayed stem-loop structures, but these structures either were not found conserved in homologous sequences in other *Borrelia *species, or the nt sequence identity was too high and thus the structures did not show base-pair changes. These were discarded. The criteria for potential RNA identification were as follows: 1) presence of the sequence in three or more different plasmid regions and/or two or more *Borrelia *species, 2) presence of a conserved stem loop with at least 9 contiguous base-pairs, 3) two or more compensatory base changes that maintain a stem, 4) in some cases, the presence of conserved looped out or bulged positions.

RNA secondary structure modeling of repeat nt sequences was performed with the Zuker and Turner mfold, version 3.2 [28,29]. Parameters used were: default window parameter, maximum interior/bulge loop size = 30, Maximum asymmetry of an interior/bulge loop = 30, and no limit on maximum distance between paired bases.

The RNAz Webserver: [58] of Gruber et al [30] was used to detect thermodynamically stable and evolutionarily conserved RNA secondary structures from multiple sequence alignments. The sequence alignment was ClustalW format. Default parameters were used, except for the Reading Direction set at forward.

The ClustalW2 program [59] provided by the EMBL-European Bioinformatics Institute [31] was used for amino acid and nucleotide sequence alignments. Parameters were as set on the EMBL-EBI web page: [32].

Random sequence analyses as described for Sequence #2 were performed using the Stothard mutagenesis program on webpage: [60].

## Abbreviations

nt: nucleotide; aa: amino acid; Ba lpX: *B. afzelii PKo *plasmid lpX; Bb lpX: *B. burgdorferi str. B31 *plasmid lpX; Bg lpX: *B. garinii PB *plasmid lpX; CRASP-1: complement regulator-acquiring surface protein_1; MITEs: miniature inverted repeat transposable elements.

## Supplementary Material

Additional file 1 **Results of RNAz analysis of Sequence** #1. The top table summarizes the sequence input and RNA structure properties. The middle diagram shows the base pairs formed between five of the repeat sequences as well as the consensus sequence. The predicted RNA secondary structure is shown at the bottom. Click here for file

Additional file 2 **Alignment of nucleotide sequences from eleven plasmid sequences related to *B. afzelii PKo *lp34 Sequence** #2. The alignment shows invariant positions as well base substitutions and deletions. Click here for file

Additional file 3 **RNA secondary structure models of Sequence #2-related nucleotide sequences from different plasmids.** Stem loop structures are highly conserved between sequences displaying base substitutions and deletions. Click here for file

Additional file 4 **Amino acid sequence alignment of CRASP-1 (Ba_lp54_mmsa_71_experimentally determined) and related loci BAPKO_2065-2070.** The alignment shows conserved and modified amino acid positions. Click here for file

Additional file 5 **RNA secondary structure models of Sequence #3 nt sequences.** Secondary structure models show two conserved stem loops. Click here for file

Additional file 6 **Alignment of Sequence #4 and related nucleotide sequences.** Alignment shows major changes in nucleotide sequences from positions 53–122. Click here for file

Additional file 7 **RNA secondary structure models of Sequence #4 and related sequences.** Conservation of overall secondary structure is maintained in the presence of major changes in nucleotide sequence. Click here for file

Additional file 8 **RNA secondary structure model of Sequence #5 from plasmid Bb lp54.** Three stem loops are depicted that are conserved in related sequences. Click here for file

Additional file 9 **RNAz analysis of Sequence #5.** Conserved RNA secondary structure parameters are shown. Click here for file
