# Supplementary material for: Intergenic regions of Borrelia plasmids contain phylogenetically conserved RNA secondary structure motifs
Source: BMC Genomics. 2009 Mar 6;10:101. doi: 10.1186/1471-2164-10-101 (PMC2674063; doi:10.1186/1471-2164-10-101)
Supplement: Additional file 1 — Results of RNAz analysis of Sequence #1. The top table summarizes the sequence input and RNA structure properties. The middle diagram shows the base pairs formed between five of the repeat sequences as well as the consensus sequence. The predicted RNA secondary structure is shown at the bottom. [file 1471-2164-10-101-S1.doc]

Additional file 1. Results of RNAz analysis of Sequence #1. The program of Gruber et al [30] was used (website: (<http://rna.tbi.univie.ac.at/cgi-bin/RNAz.cgi>). The top table summarizes the sequence input and RNA structure properties. MFW is the minimum free energy. The middle diagram shows the base pairs formed between five of the repeat sequences as well as the consensus sequence. The predicted RNA secondary structure is shown at the bottom. The colors are indicative of the compatibility of forming pairs as described in the webpage stating: Help topics, understanding the output, graphic representation. The color code for base pairs is shown in additional file 8.
