# Supplementary material for: Intergenic regions of Borrelia plasmids contain phylogenetically conserved RNA secondary structure motifs
Source: BMC Genomics. 2009 Mar 6;10:101. doi: 10.1186/1471-2164-10-101 (PMC2674063; doi:10.1186/1471-2164-10-101)
Supplement: Additional file 2 — Alignment of nucleotide sequences from eleven plasmid sequences related to B. afzelii PKo lp34 Sequence #2. The alignment shows invariant positions as well base substitutions and deletions. [file 1471-2164-10-101-S2.doc]

Additional file 2: Alignment of nucleotide sequences from eleven plasmid sequences related to *B. afzelii PKo* lp34 Sequence #2. The EMBL-EBI CLUSTALW 2.0.8 multiple sequence alignment program [31, 32] was used for alignment. Two putative paralogous sequences are found in plasmid Ba lp60 marked Ba lp60-a and Ba lp60-b. Adenosine residues are colored red, all other residues are green. Colors are for ease of viewing. The EMBL-EBI CLUSTALW 2.0.8 multiple sequence alignment program was used. A star (*) denoted invariant positions.
