# Supplementary figures and images for: Intergenic regions of Borrelia plasmids contain phylogenetically conserved RNA secondary structure motifs
Source: BMC Genomics. 2009 Mar 6;10:101. doi: 10.1186/1471-2164-10-101 (PMC2674063; doi:10.1186/1471-2164-10-101)

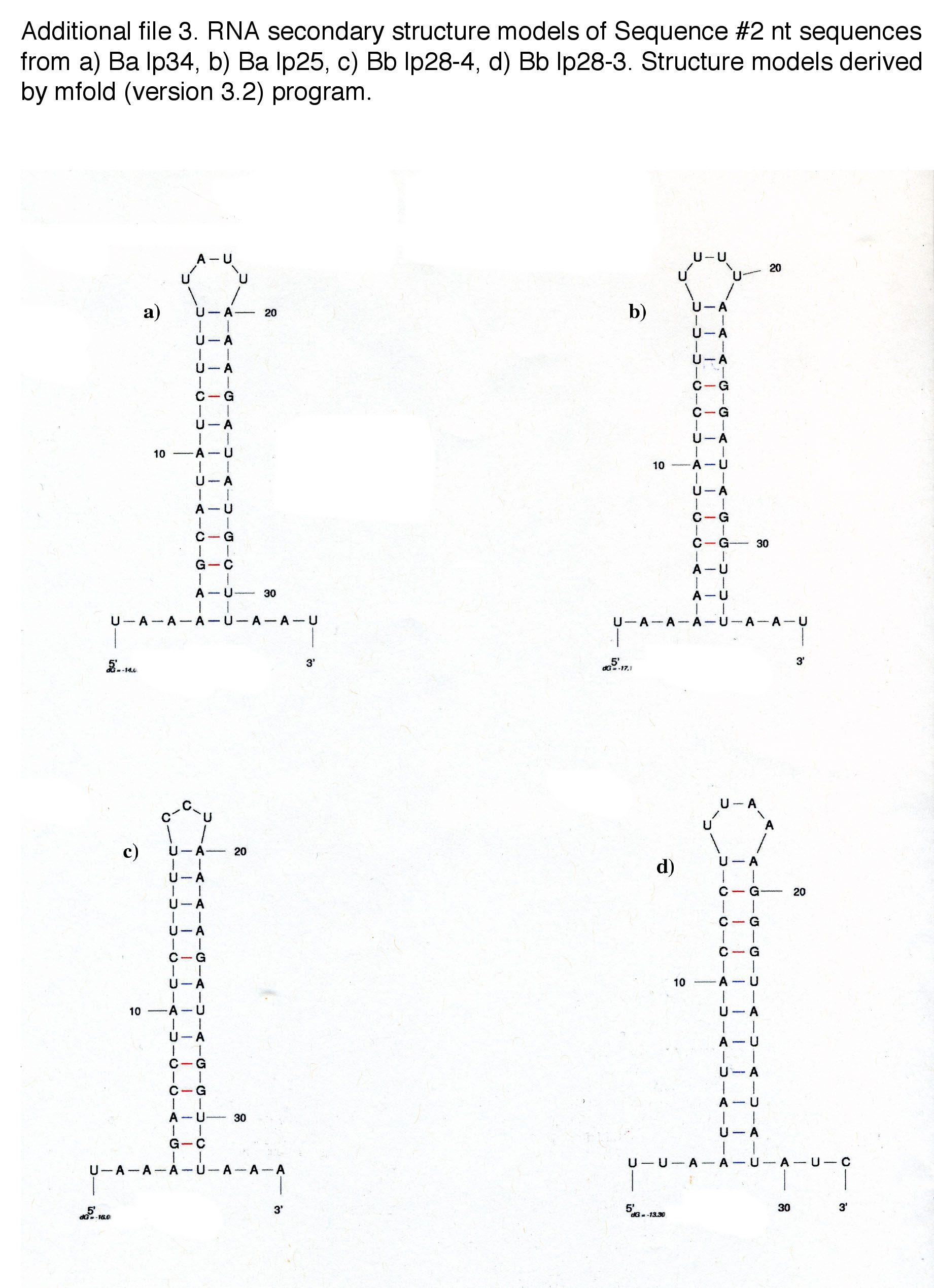

Supplement: Additional file 3 — RNA secondary structure models of Sequence #2-related nucleotide sequences from different plasmids. Stem loop structures are highly conserved between sequences displaying base substitutions and deletions. [file 1471-2164-10-101-S3.jpeg]
