# Supplementary material for: Intergenic regions of Borrelia plasmids contain phylogenetically conserved RNA secondary structure motifs
Source: BMC Genomics. 2009 Mar 6;10:101. doi: 10.1186/1471-2164-10-101 (PMC2674063; doi:10.1186/1471-2164-10-101)
Supplement: Additional file 4 — Amino acid sequence alignment of CRASP-1 (Ba_lp54_mmsa_71_experimentally determined) and related loci BAPKO_2065-2070. The alignment shows conserved and modified amino acid positions. [file 1471-2164-10-101-S4.doc]

Additional file 4. Top: Amino acid sequence alignment of CRASP-1 (Ba_lp54_mmsa_71_experimentally determined) and related loci BAPKO_2065-2070. Alignment is according to EMBL-EBI CLUSTALW 2.0.8 multiple sequence alignment program [31, 32]. Symbols under columns: (*) denotes invariant amino acid positions, (:) denotes conserved substitutions, (.) denotes semi-conserved substitutions. Color code as described in Figure 3.

Bottom: Phylogram of CRASP-1 and related loci BAPKO_2065-2070. Branch lengths are considered proportional to evolutionary changes. Numbers next to loci names denote branch distances.
