# Supplementary material for: Intergenic regions of Borrelia plasmids contain phylogenetically conserved RNA secondary structure motifs
Source: BMC Genomics. 2009 Mar 6;10:101. doi: 10.1186/1471-2164-10-101 (PMC2674063; doi:10.1186/1471-2164-10-101)
Supplement: Additional file 5 — RNA secondary structure models of Sequence #3 nt sequences. Secondary structure models show two conserved stem loops. [file 1471-2164-10-101-S5.doc]

Additional file 5. RNA secondary structure models of Sequence #3 nt sequences downstream from a) BAPKO_2065 and b) CRASP_1 from Ba lp54 *mmsa7*. Structure models derived by mfold (version 3.2) program [28, 29].
