# Supplementary material for: Intergenic regions of Borrelia plasmids contain phylogenetically conserved RNA secondary structure motifs
Source: BMC Genomics. 2009 Mar 6;10:101. doi: 10.1186/1471-2164-10-101 (PMC2674063; doi:10.1186/1471-2164-10-101)
Supplement: Additional file 6 — Alignment of Sequence #4 and related nucleotide sequences. Alignment shows major changes in nucleotide sequences from positions 53–122. [file 1471-2164-10-101-S6.doc]

Additional file 6. Alignment of nucleotide sequences (Sequence #4) from plasmid sequences from *B. afzelii PKo* and *B. garinii PB* and *B. burgdorferi B31*. Positions on plasmid sequences are also shown. Adenosine residues are colored red, all other residues are green. Colors are for ease of viewing. The EMBL-EBI CLUSTALW 2.0.8 multiple sequence alignment program was used. A star (*) denoted invariant positions.
