# Supplementary material for: Intergenic regions of Borrelia plasmids contain phylogenetically conserved RNA secondary structure motifs
Source: BMC Genomics. 2009 Mar 6;10:101. doi: 10.1186/1471-2164-10-101 (PMC2674063; doi:10.1186/1471-2164-10-101)
Supplement: Additional file 7 — RNA secondary structure models of Sequence #4 and related sequences. Conservation of overall secondary structure is maintained in the presence of major changes in nucleotide sequence. [file 1471-2164-10-101-S7.doc]

Additional file 7. RNA secondary structure models of Sequence #4 122 nt sequence from plasmids a) Ba lp60, b) Bb lp54, and c) Bg lp54. Stem loops 1, 2, and 3 are depicted. Structure models derived by mfold (version 3.2) program [28, 29].
