# Supplementary material for: Intergenic regions of Borrelia plasmids contain phylogenetically conserved RNA secondary structure motifs
Source: BMC Genomics. 2009 Mar 6;10:101. doi: 10.1186/1471-2164-10-101 (PMC2674063; doi:10.1186/1471-2164-10-101)
Supplement: Additional file 8 — RNA secondary structure model of Sequence #5 from plasmid Bb lp54. Three stem loops are depicted that are conserved in related sequences. [file 1471-2164-10-101-S8.doc]

Additional file 8. RNA secondary structure model of Sequence #5 150 nt sequence from plasmid Bb lp54. Stem loops 1, 2, and 3 are depicted. Structure models derived by mfold (version 3.2) program.
