# Supplementary material for: Intergenic regions of Borrelia plasmids contain phylogenetically conserved RNA secondary structure motifs
Source: BMC Genomics. 2009 Mar 6;10:101. doi: 10.1186/1471-2164-10-101 (PMC2674063; doi:10.1186/1471-2164-10-101)

Additional file 9. RNAz analysis of Sequence #5. Data taken from the RNAz website designed to determine thermodynamically stable secondary structures [30]. MFW is the minimum free energy. The prediction is that of an RNA structure with a mean z-score of –6.04. The secondary structure shown below is highly similar to the secondary structure with an m-fold analysis of Sequence #5, i.e., [Structure 2](http://mfold.bioinfo.rpi.edu/results/11/08Dec09-11-26-37/08Dec09-11-26-37.out.html#STRUCTURE_2): Initial delta G = -52.70 kcal/mol, (data not shown). The secondary structure shown in the figure of additional file 7 represents the first structure shown by m-fold analysis, [Structure 1](http://mfold.bioinfo.rpi.edu/results/11/08Dec09-11-26-37/08Dec09-11-26-37.out.html#STRUCTURE_1) : Initial delta G = -53.30 kcal/mol. The color code for base pairs shown below is from the RNAz website.

| **Location** | **0 – 150** |
| --- | --- |
| **Length** | **150** |
| **RNAz 1.0 Sequences** | **3** |
| **Columns** | **150** |
| **Reading direction** | **forward** |
| **Mean pairwise identity** | **96.67** |
| **Mean single sequence MFE** | **-47.13** |
| **Consensus MFE** | **-45.75** |
| **Energy contribution** | **-45.53** |
| **Covariance contribution** | **-0.22** |
| **Combinations/Pair** | **1.06** |
| **Mean z-score** | **-6.04** |
| **Structure conservation index** | **0.97** |
| **SVM decision value** | **1.01** |
| **SVM RNA-class probability** | **0.899096** |
| **Prediction** | **RNA** |


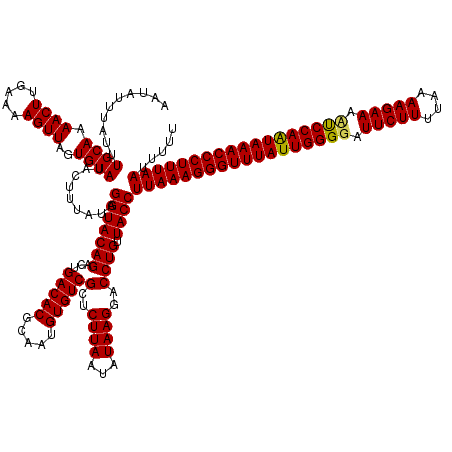


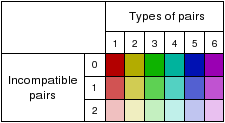

Supplement: Additional file 9 — RNAz analysis of Sequence #5. Conserved RNA secondary structure parameters are shown. [file 1471-2164-10-101-S9.doc]
